# Antifungal Activity of 5-Fluorouridine Against *Candida albicans* and *Candida parapsilosis* Based on Virulence Reduction

**DOI:** 10.3390/molecules30132735

**Published:** 2025-06-25

**Authors:** Ewa Lenarczyk, Damian Oleksiak, Monika Janeczko

**Affiliations:** 1 Doctoral School, The John Paul II Catholic University of Lublin, al. Racławickie 14, 20-950 Lublin, Poland; ewa.lena999@gmail.com; 2 Department of Molecular Biology, Faculty of Medicine, The John Paul II Catholic University of Lublin, Konstantynów 1i, 20-708 Lublin, Poland; 3 Experimental Research Center, Faculty of Medicine, The John Paul II Catholic University of Lublin, Konstantynów 1i, 20-708 Lublin, Poland; damian.oleksiak@kul.pl

**Keywords:** antifungals, hemolysis, in vitro activity, nucleosides, zebrafish

## Abstract

This study aims to explore the potential repurposing of 5-fluorouridine (5-FUrd) as an antifungal agent against *Candida* species. We evaluated the responses of nine reference species of *Candida* spp. and one hundred clinical isolates of *C. albicans* to 5-FUrd using the broth microdilution method. Additionally, we assessed the effect of 5-FUrd on selected virulence factors, including biofilm formation, cell adhesion, dimorphism, hydrolase secretion, and hemolytic activity, in the two most sensitive *Candida* species, *C. albicans* and *C. parapsilosis*. The frequency of spontaneous mutations occurring in these two *Candida* species under the influence of 5-FUrd was also determined. Finally, we examined the cytotoxic properties of 5-FUrd against human erythrocytes and zebrafish embryos. Our results demonstrated that 5-FUrd exhibits antifungal activity in vitro, inhibits biofilm formation, suppresses hyphal growth, reduces cell surface hydrophobicity, eradicates mature biofilms, and decreases the secretion of extracellular proteinases and hemolytic activity in *C. albicans* and *C. parapsilosis* cells. The overall mutation frequency under the selective pressure of 5-FUrd ranged from 2 × 10^−5^ to 1.2 × 10^−4^ per species. Notably, the exposure to 5-FUrd did not induce significant toxic effects on human erythrocytes or zebrafish embryos. This study highlights the potential clinical application of 5-FUrd as an anti-*Candida* agent.

## 1. Introduction

Nucleosides, the fundamental components of nucleic acids, play a key role in many crucial biochemical processes in all organisms. Chemically modified nucleoside analogs mimic their endogenous counterparts, exploiting cellular metabolism and becoming incorporated into both DNA and RNA. This property, which is related to their ability to inhibit enzymes essential for the replication of pathogens and the proliferation of cancer cells, makes nucleoside analogs effective drugs in the treatment of various conditions, mainly viral, bacterial, and cancer diseases [1,2,3].

In recent years, there has been increasing interest in nucleosides as potential agents for the treatment of fungal infections, especially in light of the rising resistance to conventional antifungal drugs [4,5]. Over the last decade, the incidence of fungal infections has increased, particularly among immunocompromised individuals or those hospitalized with severe illnesses. These infections often arise as secondary complications in patients with such conditions as cancer, AIDS, asthma, diabetes, organ transplants, chemotherapy, and corticosteroid treatment. Among fungal infections, *Candida*, *Aspergillus*, *Cryptococcus*, and *Pneumocystis* are major risk factors worldwide due to the severity and higher incidence of the disease [6]. *Candida* species represent an important source of yeast infections. In 2022, the World Health Organization (WHO) released a list of fungal priority pathogens categorized into three priority groups. In this classification, *C. auris* (currently named *Candidozyma auris*) and *C. albicans* are designated as critical pathogens. *C. glabrata* (currently named *Nakaseomyces glabratus*), *C. parapsilosis*, and *C. tropicalis* are classified into the high-priority group, and *C. krusei* (currently named *Pichia kudriavzevii*) is placed in the medium-priority group [7]. These opportunistic microorganisms are not only the most common cause of superficial vaginal or mucosal oral infections but can also enter the bloodstream leading to hard-to-treat deep-tissue infections [8].

Currently, antifungal treatments for invasive mycoses are limited to five major classes of compounds: echinocandins, imidazoles, triazoles, polyenes, and nucleoside analogs [9]. Among antifungal nucleosides, flucytosine, whose active compound is 5-fluorocytosine (5-FC), a fluorinated pyrimidine derivative, is widely used. Flucytosine belongs to the group of antifungal antimetabolites. It penetrates fungal cells via cytosine deaminase, converting to fluorouracil, which is then incorporated into RNA, thereby inhibiting protein synthesis. Mammalian cells lack this enzyme, rendering flucytosine non-toxic to humans. Additionally, flucytosine is metabolized into fluorodeoxyuridine, which disrupts DNA synthesis and impairs fungal cell division. Flucytosine is effective against species from the genera *Candida*, *Cryptococcus*, *Cladosporium*, *Phialophora*, *Cyberlindnera*, *Debaryomyces*, *Diutina*, *Rhodotorula*, *Saccharomyces*, and *Aspergillus*. In combination with the polyene agent amphotericin B, flucytosine is a reliable treatment for resistant *Candida* infections and cryptococcal meningitis [10]. However, its clinical utility is limited by the frequent emergence of both primary and acquired resistance, particularly when used as monotherapy. Primary resistance to 5-FC is found in less than 5% of all *Candida* species, except for *C. krusei*, where resistance is observed in up to 35% of isolates. Resistance mechanisms are complex and can involve mutations or loss of function in any of the three key enzymes: FCY2 (cytosine permease), FCY1 (cytosine deaminase), or FUR1 (uracil phosphoribosyltransferase). FCY2 is responsible for the active transport of flucytosine into fungal cells, while FCY1 and FUR1 convert it into its toxic metabolite, 5-fluoro-uridylate. Additionally, increased endogenous pyrimidine production can help fungi circumvent the toxic effects of antifungal agents [11].

Given the growing burden of fungal diseases and antimicrobial resistance, new therapeutic options are urgently needed. Among the most innovative approaches is de novo drug discovery, which involves the identification of both synthetic compounds and natural products derived from various sources, such as microbial strains, plants, algae, endophytic fungi, and marine fungi. Importantly, antifungal therapies in current clinical development are being designed based on a broad range of strategies, including the targeting of known molecular pathways (e.g., cell wall synthesis inhibitors like fosmanogepix or nikkomycin Z), novel or previously unexploited targets such as calcineurin, Hsp90, lipid biosynthesis, and kinases, as well as the development of entirely new molecular scaffolds (e.g., tetrazoles like oteseconazole and VT-1598, BSG005, encochleated amphotericin B, rezafungin, or AM-2-19). In addition, another promising strategy is also drug repurposing and re-evaluating existing non-antifungal compounds for potential antifungal activity, thus bypassing the high costs and lengthy timelines of traditional drug development [12,13].

Since nucleoside analogs are a class of drugs which are important from a clinical perspective and show promise in the treatment of mycoses, in this study, we used 5-fluorouridine (riboside of 5-fluoro-2,4(1H, 3H)-pyrimidinedione, 5-FUrd), i.e., a fluoropyrimidine nucleoside analog, to evaluate its efficacy against *Candida* species. 5-FUrd is a cell-permeable modified RNA precursor and a prodrug of 5-fluorouracil (5-FU) [14]. Previous studies have demonstrated its antifungal activity against *C. albicans* attributed to inhibition of thymidylate kinase (CaTMPK), a key enzyme in the dTTP biosynthesis pathway. CaTMPK catalyzes the phosphorylation of dTMP to dTDP, which is subsequently converted to dTTP by nucleoside diphosphate kinase (NDPK). Several factors support the potential of 5-FUrd as a selective antifungal agent: (i) Structural differences exist between CaTMPK and its human counterpart (hTMPK), particularly in the Ca-loop, a unique surface-exposed catalytic element that drives hyperactivity in CaTMPK. The deletion of this loop significantly impairs *C. albicans* growth, identifying it as a promising target for fungal-specific inhibitors. (ii) The catalytic efficiency of CaTMPK is 15-fold higher than that of hTMPK. (iii) CaTMPK exhibits strong activity in converting dUMP to dUDP and 5-FdUMP to 5-FdUDP, enhancing the cytotoxicity of 5-FUrd. (iv) 5-FUrd is effective against *C. albicans* strains resistant to both 5-FC and azoles due to differences in their metabolic activation pathways. 5-FUrd enters *C. albicans* via uridine permease (Fui1) and is phosphorylated by uridine kinase (Urk1) to 5-FUMP, bypassing the resistance mechanisms that impair 5-FC activation [15].

Initial studies on the antifungal activity of 5-FUrd focused exclusively on *C. albicans*, a major opportunistic fungal pathogen and a widely used model organism in antifungal research [15]. Encouraged by the promising results obtained in this species, we substantially expanded the scope of our investigation to include additional *Candida* species and a broad collection of clinical isolates of *C. albicans*. This comprehensive approach aimed to assess the wider antifungal spectrum of 5-FUrd and to determine whether the observed effects could be replicated in other clinically relevant *Candida* species, particularly those with distinct susceptibility profiles and virulence traits. In parallel, the study was extended beyond conventional susceptibility testing to include key virulence-associated features, such as adhesion, biofilm formation, dimorphism, hydrolase secretion, hemolytic activity, and cell surface hydrophobicity, in two species found to be particularly sensitive to 5-FUrd: *C. albicans* and *C. parapsilosis*. These pathogenicity-related factors are known to play a critical role in infection persistence and resistance to treatment. Collectively, this expanded investigation provides an in-depth evaluation of 5-FUrd as a promising antifungal candidate, with dual potential for both growth inhibition and attenuation of fungal virulence. Additionally, we assessed the frequency of spontaneous mutations in *C. albicans* and *C. parapsilosis* under drug pressure and evaluated 5-FUrd cytotoxicity using human erythrocytes and a zebrafish model.

## 2. Results

### 2.1. Antifungal Effects of 5-Fluorouridine Against Planktonic Cells of Candida Species

To evaluate the antifungal activity of 5-FUrd against planktonic cells of nine reference *Candida* species, minimum inhibitory concentrations (MICs) and minimum fungicidal concentrations (MFCs) were determined. The results are summarized in Table 1. The MIC and MFC values ranged from 0.2 μg/mL to 51.2 μg/mL. Among the tested species, *C. albicans* and *C. parapsilosis* exhibited the highest susceptibility to 5-FUrd, while *C. auris* demonstrated resistance within the tested concentration range. Based on the MFC/MIC ratio, the mechanism of action of 5-FUrd was determined. An antifungal agent is considered a priori fungicidal if the ratio of MFC to MIC does not exceed 4. An antifungal agent with a ratio higher than 4 is considered fungistatic. The MFC/MIC ratio for all the microorganisms was extremely low, indicating that 5-FUrd is fungistatic and fungicidal for these *Candida* species.

**Figure 1:**
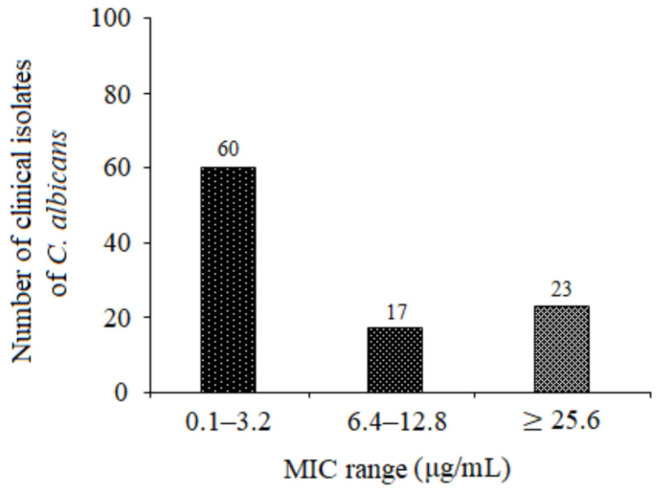
Distribution of 5-FUrd MICs for clinical *C. albicans* strains.

In order to study the antifungal activity of 5-FUrd against clinical *Candida* strains, a large panel of 100 *C. albicans* strains was tested, and 5-FUrd MICs were determined. We reported here the efficacy of this compound against a collection of 60 clinical *C. albicans* strains, with MICs ranging from 0.1 μg/mL to 3.2 μg/mL. In the case of 17 of the strains, the MICs amounted to 6.4 μg/mL or 12.8 μg/mL. Conversely, the 5-FUrd MICs obtained for 23 strains were ≥25.6 μg/mL (Figure 1).

Given the pronounced activity of 5-FUrd against *Candida albicans* and *Candida parapsilosis* (Table 1), subsequent experiments were focused on these two species.

### 2.2. Effect of 5-FUrd on Metabolic Activity and Biomass of Biofilms

**Figure 2:**
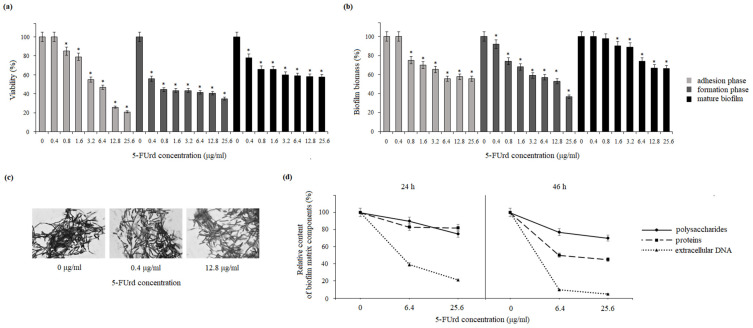
Influence of 5-fluorouridine on *C. albicans* biofilms: (**a**) reduction in biofilm cell viability assessed by the MTT assay; (**b**) decrease in total biomass based on crystal violet staining; (**c**) microscopic visualization of biofilm architecture following the treatment; (**d**) alterations in the relative content of key extracellular matrix components (proteins, polysaccharides, and extracellular DNA). Data are presented as percentages relative to the untreated control (set to 100%). * *p* < 0.05 indicates statistically significant differences compared to the control.

To assess the effect of 5-FUrd on biofilms formed by *C. albicans* and *C. parapsilosis*, two complementary methods were employed: the MTT assay and crystal violet (CV) staining. The MTT assay was used to evaluate cell viability within the biofilm during the adhesion, formation, and mature phases. 5-FUrd was applied at concentrations ranging from 0.4 μg/mL to 25.6 μg/mL. The viability of *C. albicans* cells in the biofilm during the initial growth phase (adhesion phase) decreased to 85–22% under the influence of 5-FUrd, compared to the untreated cells (control). The exposure to 5-FUrd significantly reduced cell viability during 24 h biofilm formation to 55–34%, compared to the control (untreated cells). Furthermore, the compound was effective against mature biofilm, reducing its metabolic activity to 75–60% (Figure 2a). The crystal violet staining also confirmed the reducing effect of 5-FUrd on the *C. albicans* biofilm biomass. During the cell adhesion phase, the yeast cell biomass decreased to 76–58% under the influence of the compound at 0.8–25.6 μg/mL. In the biofilm maturation phase, a biomass reduction of 90–35% was observed under the compound pressure at 0.4–25.6 μg/mL. Moreover, the treatment of mature biofilm with 5-FUrd at the concentrations of 1.6–25.6 μg/mL resulted in its eradication and a reduction in cell biomass up to 89–63%, compared to the control group (Figure 2b). The microscopic analysis further demonstrated a concentration-dependent disruption of biofilm architecture. The exposure to 0.4 μg/mL and 12.8 μg/mL of 5-FUrd resulted in visibly fewer cells and a loosened biofilm structure, in contrast to the dense, compact structure observed in the untreated samples (Figure 2c).

**Figure 3:**
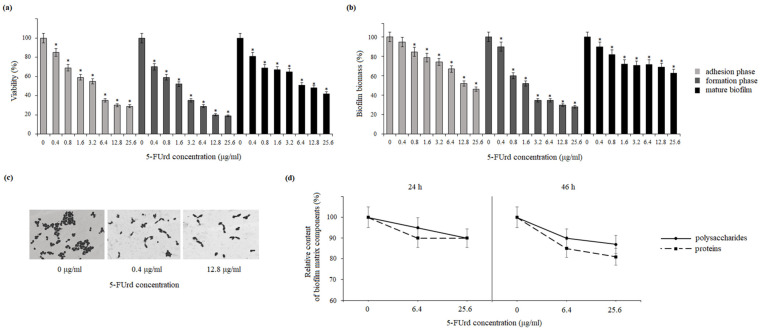
Influence of 5-fluorouridine on *C. parapsilosis* biofilms: (**a**) reduction in biofilm cell viability at various stages of development (adhesion, formation, and maturation); (**b**) decrease in total biomass across different biofilm growth phases; (**c**) microscopic visualization of biofilm architecture following the treatment; (**d**) alterations in the relative abundance of key extracellular matrix components (proteins and polysaccharides). Data are expressed as percentages relative to the untreated control (set to 100%). * *p* < 0.05 indicates statistically significant differences compared to the control.

Similar anti-biofilm effects were observed for *C. parapsilosis*. The MTT assay showed that 5-FUrd significantly decreased cell viability across all the biofilm developmental phases. During the adhesion phase, 5-FUrd (0.4–25.6 μg/mL) reduced the viability to 86–31%. In the biofilm formation phase, the viability dropped to 49–18%. For mature biofilms, metabolic activity was reduced to 82–40% within the same concentration range (Figure 3a). The assessment of biofilm biomass using CV staining showed that 5-FUrd (0.8–25.6 μg/mL) reduced the biomass during the adhesion phase by 88–47%. During the 24 h biofilm development phase, the biomass was reduced to 91–30%, depending on the concentration. Furthermore, 5-FUrd was able to penetrate mature *C. parapsilosis* biofilms, resulting in a biomass decrease of 90–61%, compared to the controls (Figure 3b). The microscopic imaging of *C. parapsilosis* biofilms exposed to 0.4 μg/mL and 12.8 μg/mL of 5-FUrd revealed substantial cell loss and structural disruption, compared to the untreated biofilms (Figure 3c).

### 2.3. Effect on Extracellular Polymeric Substances (EPS)

The impact of 5-FUrd on the extracellular polymeric substance (EPS) matrix of *C. albicans* and *C. parapsilosis* biofilms was evaluated after 24 and 48 h of growth in the presence of 6.4 μg/mL and 25.6 μg/mL of the compound. In the *C. albicans* biofilm matrix, the polysaccharide content decreased to 92% and 80% after 24 h of incubation and to 80% and 78% after 48 h, compared to the controls. The protein content decreased to 30% after 24 h and to 60% after 48 h, respectively. A significant decrease in the concentration of extracellular DNA due to the influence of 5-FUrd was evident after 24 h of biofilm growth, with a reduction to 30% and 40%, compared to the untreated cells. This decrease intensified after 48 h, reaching 30% and 20%, respectively. The proteins in the matrix of mature *C. albicans* biofilms were also reduced to 80% and 78% after 24 h of biofilm formation and to 52% and 49% after 48 h of biofilm growth. In contrast, compared to the control biofilms without 5-FUrd, the extracellular DNA content was reduced to 38% and 20% after 24 h and to 8% and 4% after 48 h of growth in the presence of 6.4 μg/mL and 25.6 μg/mL of 5-FUrd, respectively (Figure 2d). In the matrix of *C. parapsilosis* biofilms, only the levels of polysaccharides and proteins were determined due to the lack of extracellular DNA. The relative content of polysaccharides gently decreased to 95% and 91% after 24 h of growth and to 89% and 87% after 48 h of incubation in the presence of 5-FUrd at the concentrations of 6.4 μg/mL and 25.6 μg/mL, respectively. A decrease in the protein concentration was also observed in the biofilm culture in identical conditions. In this case, the content of proteins declined to 90% and 89% and to 84% and 82% after the longer 48 h incubation (Figure 3d).

### 2.4. Effect on Cell Surface Hydrophobicity

**Figure 4:**
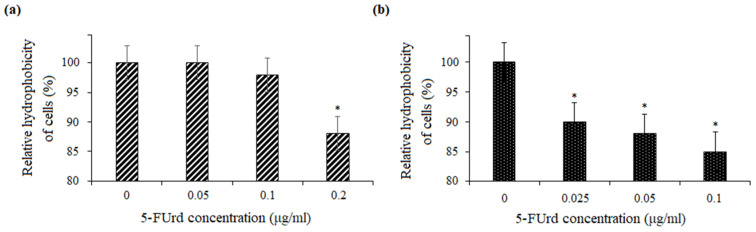
Effect of 5-FUrd on cell surface hydrophobicity (CSH) of *Candida* species: (**a**) reduction in CSH of *C. albicans* ATCC 10231; (**b**) decrease in CSH of *C. parapsilosis* ATCC 22099. Data are expressed relative to the untreated control (set to 100%). * *p* < 0.05 indicates a statistically significant difference compared to the control.

The effect of 5-FUrd on the cell surface hydrophobicity (CSH) of *Candida* species was measured based on the percentage of cell adsorption to octane. The results showed that cell hydrophobicity was significantly compromised and decreased following the exposure of *C. albicans* to 0.2 μg/mL and *C. parapsilosis* to 0.025 μg/mL, 0.05 μg/mL, and 0.1 μg/mL of 5-FUrd. The CSH of *C. albicans* was reduced to 88%, compared to the untreated yeast cells (control) (Figure 4a). Similarly, the CSH of *C. parapsilosis* decreased within the range of 85–90%, compared to the control (cells without 5-FUrd) (Figure 4b).

### 2.5. Influence of 5-FUrd on Morphological Changes in Candida

**Figure 5:**
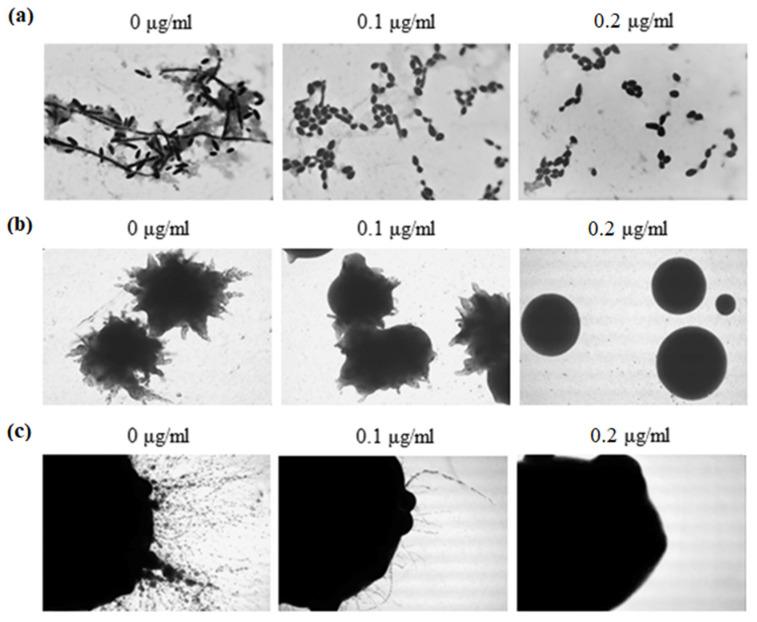
Representative microscopic images of *C. albicans* ATCC 10231 cultured in RPMI-1640 medium supplemented with 10% FBS: (**a**) cells grown in liquid medium after 10 h of incubation at 37 °C; (**b**) whole colonies formed on solid medium after 24 h of growth; (**c**) morphology of colony edges. The cultures were treated with 5-FUrd at 0.1 µg/mL and 0.2 µg/mL and compared to the untreated controls (0 µg/mL).

To investigate the effect of 5-FUrd on the morphology of cells and colonies of *C. albicans* and *C. parapsilosis*, yeast cells were cultured in both liquid and solid RPMI-1640 media supplemented with 10% FBS. Our results demonstrated that 5-FUrd negatively affected hyphal formation in *C. albicans* in a dose-dependent manner both in liquid cultures and in colonies grown on solid media (Figure 5). The microscopic analysis performed after 10 h of growth in liquid medium revealed that cells cultured in the control conditions (without 5-FUrd) exhibited typical morphology, including hyphae, pseudohyphae, and yeast forms. In contrast, cultures treated with 5-FUrd showed fewer cells attached to the coverslip, and the predominant morphology was yeast-form cells (Figure 5a). Similarly, a notable reduction in hyphal structures was observed in *C. albicans* colonies grown on solid media in the presence of 5-FUrd, compared to the untreated control colonies (Figure 5b,c).

**Figure 6:**
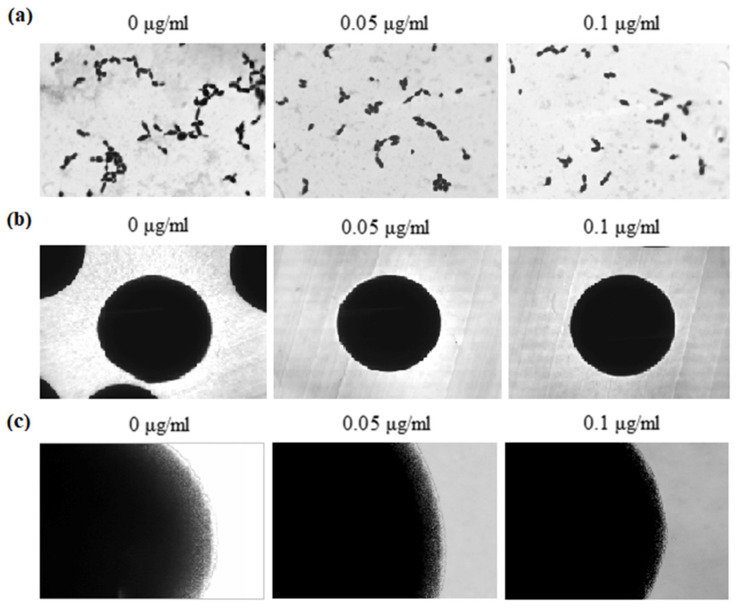
Representative microscopic images of *C. parapsilosis* ATCC 22099 cultured in RPMI-1640 medium supplemented with 10% FBS: (**a**) cells grown in liquid medium after 10 h of incubation at 37 °C; (**b**) whole colonies formed on solid medium after 24 h of growth; (**c**) morphology of colony edges. The cultures were treated with 5-FUrd at 0.05 µg/mL and 0.1 µg/mL and compared to the untreated controls (0 µg/mL).

To assess the effect of 5-FUrd on *C. parapsilosis* morphology in liquid medium, the cells were cultured in the presence of the compound at concentrations of 0.1 μg/mL and 0.05 μg/mL. The microscopic images revealed a significant reduction in the cell number and marked inhibition of pseudohyphal formation, with only sporadic pseudohyphae observed (Figure 6a). In contrast, no morphological differences were observed between the 5-FUrd-treated and untreated *C. parapsilosis* colonies grown on solid media, as all colonies appeared smooth and lacked pseudohyphal structures (Figure 6b,c).

### 2.6. Activity of 5-FUrd Against Hydrolase Secretion and Hemolytic Factor Production

To evaluate the effect of 5-FUrd on the secretion of phospholipases, egg yolk-supplemented media were used, and the precipitation zones around the colonies were measured. As shown in Table 2, the Pz values of *C. albicans* exposed to three different subtherapeutic concentrations of 5-FUrd were comparable to those of the positive control (untreated cells). Similarly, the treatment of *C. parapsilosis* cells with 0.025 µg/mL, 0.05 µg/mL, and 0.1 µg/mL of 5-FUrd did not reduce their phospholipase activity. No statistically significant differences in Pz values were observed between the positive control and the treated samples. The negative control strain, *C. glabrata*, exhibited no phospholipase activity (Table 2).

**Figure 7:**
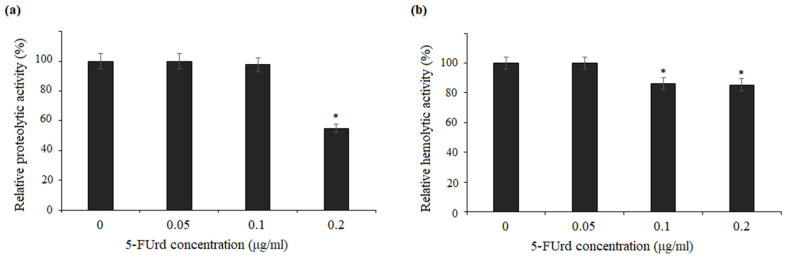
Effect of 5-FUrd on the extracellular enzymatic activities of *C. albicans* ATCC 10231: (**a**) relative proteolytic activity of cells treated with increasing concentrations of 5-FUrd expressed as a percentage of the control (untreated cells, set to 100%); (**b**) hemolytic activity in culture supernatants following the 5-FUrd treatment expressed relative to the untreated control. * *p* < 0.05 significance compared to the control group (untreated cells) set to 100%.

**Figure 8:**
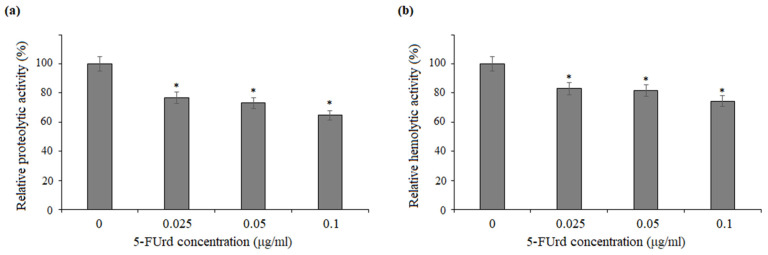
Effect of 5-FUrd on the extracellular enzymatic activities of *C. parapsilosis* ATCC 22099: (**a**) relative proteolytic activity of cells treated with increasing concentrations of 5-FUrd expressed as a percentage of the control (untreated cells, set to 100%); (**b**) hemolytic activity in culture supernatants following the 5-FUrd treatment expressed relative to the untreated control. * *p* < 0.05 significance compared to the control group (untreated cells) set to 100%.

A casein assay was used to determine the effect of 5-FUrd on the secretion and activity of proteinases from *C. albicans* and *C. parapsilosis*. The decrease in proteolytic activity after the treatment with the tested compound was expressed as a percentage of the control (untreated cells). As shown in Figure 7a, 5-FUrd reduced the proteolytic activity of *C. albicans* to about 55% at the concentration of 0.2 µg/mL. Correspondingly, as shown in Figure 8a, *C. parapsilosis* cells exposed to 5-FUrd at the concentrations of 0.025 µg/mL, 0.05 µg/mL, and 0.1 µg/mL exhibited a reduction in proteolytic activity to 77%, 73%, and 65%, respectively.

Figure 7b and Figure 8b further demonstrate that 5-FUrd decreased the hemolytic activity of *Candida* cells. In the culture supernatants of *C. albicans*, a modest decline in hemolytic activity was observed, reaching 86.1% and 85.3% of the control levels following the exposure to 0.1 µg/mL and 0.2 µg/mL of 5-FUrd, respectively (Figure 7b). Similarly, the hemolytic activity in the supernatants of *C. parapsilosis* cultures decreased to 83%, 82%, and 74.5% at the concentrations of 0.025 µg/mL, 0.05 µg/mL, and 0.1 µg/mL, respectively (Figure 8b).

### 2.7. Spontaneous Mutation Frequencies

The spontaneous mutation frequencies in *C. albicans* ATCC 10231 on SDA medium supplemented with 0.4 µg/mL of 5-FUrd ranged from 6 × 10^−5^ to 1.2 × 10^−4^ per plate. At the higher drug concentrations (0.8 µg/mL and 1.6 µg/mL), no colony growth was observed, indicating a lack of resistant mutant emergence in these conditions. For *Candida parapsilosis* ATCC 22099, the mutation frequencies on SDA plates containing 0.2 µg/mL, 0.4 µg/mL, and 0.8 µg/mL of 5-FUrd ranged from 8.8 × 10^−5^ to 1.2 × 10^−4^, 6.8 × 10^−5^ to 1.1 × 10^−4^, and 2 × 10^−5^ to 4.4 × 10^−5^ per plate, respectively. Thus, the overall mutation frequencies observed across both strains ranged from 2 × 10^−5^ to 1.2 × 10^−4^. All *C. albicans* and *C. parapsilosis* mutants exhibited a minimum fourfold increase in the MIC for 5-FUrd, compared to their respective parental strains, confirming the selection of resistant phenotypes (Table 3).

### 2.8. Toxic Effects of 5-FUrd on Zebrafish Embryos and Human Erythrocytes

**Figure 9:**
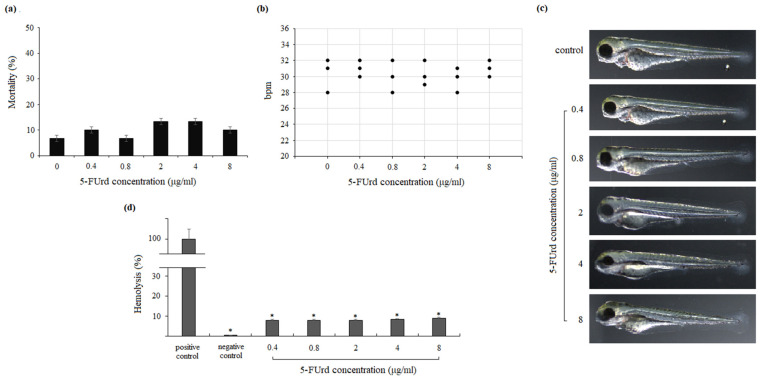
Evaluation of 5-FUrd toxicity in zebrafish embryos and human erythrocytes: (**a**) mortality of zebrafish embryos following 96 h treatments with 5-FUrd; (**b**) heart rate (beats per minute, bpm) of zebrafish embryos following 96 h treatments with 5-FUrd; (**c**) influence of 5-FUrd on the embryonic development of zebrafish; (**d**) effect of 5-FUrd on human erythrocytes. * *p* < 0.05 indicates significance compared to the positive control set to 100%.

To evaluate the potential of 5-FUrd as an antimicrobial agent, its toxicity profile was assessed using zebrafish embryos and human erythrocytes. Zebrafish embryos were exposed to 5-FUrd for 96 h, and key toxicological endpoints, i.e., mortality, heart rate, and morphological abnormalities, were monitored and recorded. Mortality was calculated as the percentage of dead embryos relative to the total number of fertilized eggs per treatment group. In the control groups, mortality remained below 10% at 96 h in all the replicates. As shown in Figure 9a, embryo mortality following the exposure to 5-FUrd at concentrations ranging from 0.4 to 8 µg/mL did not differ significantly from that of the untreated control group and did not exceed 14% in any treatment condition. The heart rate (beats per minute) at the end of the treatment was observed using a microscope and counted with a cell counter. The heart rate of the 5-FUrd-treated samples was compared with the heart rate of the control embryos (E3 medium) at 96 h. As illustrated in Figure 9b, the 5-FUrd treatment did not significantly affect the heart rate at any tested concentration. Since zebrafish is a promising model for whole-organism toxicology screening due to its rapid development, we evaluated the morphological changes in zebrafish embryos induced by 5-FUrd. As shown in Figure 9c, the experiment revealed neither morphological changes in the zebrafish embryos nor any toxic effects of the compound when used at the doses of 0.4 to 8 µg/mL over 5 days of observation.

Additionally, the toxic effects of 5-FUrd were evaluated by the induction of erythrocyte lysis. For this purpose, human erythrocytes were incubated in the presence of different concentrations of 5-FUrd. As presented in Figure 9d, 5-FUrd induced less than 10% erythrocyte lysis across all the tested concentrations (0.4–8 µg/mL), indicating low cytotoxicity toward human red blood cells and supporting the safety profile of the compound applied at antimicrobial doses.

## 3. Discussion

This study provides new insights into the antifungal potential of 5-FUrd, demonstrating its broad-spectrum activity against various *Candida* species, including clinical isolates. In addition to its growth-inhibitory effects, 5-FUrd was found to impair key virulence factors, such as biofilm formation, hyphal development, and enzymatic activity, i.e., traits that are essential for fungal pathogenicity and persistence within host environments. Notably, our evaluation of toxicity and mutagenicity offers preliminary evidence supporting a favorable safety profile and therapeutic potential.

In an era of rapidly increasing resistance to antimicrobial agents, it is extremely important to search for new antifungal substances that are effective against pathogenic fungi exhibiting resistance to conventional therapies. In this study, we investigate the effect of 5-FUrd on the growth and virulence of *Candida* species. The biological activity spectrum of 5-FUrd and its derivatives primarily includes antitumor and antiviral effects [14,16,17,18]. Previous studies have also demonstrated the antifungal activity of 5-FUrd attributed to its ability to inhibit thymidylate kinase in *C. albicans* cells [15]. Thus, 5-FUrd can be classified among nucleoside derivatives with antifungal activity, alongside such compounds as tolytoxin, tubercidin, nikkomycin, polyoxin, blasticidin S, arginomycin, mildiomycin, cordycepin, sinefungin, toyocamycin, and clavines [4,19,20,21,22,23,24]. To expand upon earlier research, we conducted studies on selected *Candida* reference strains as well as clinical isolates of *C. albicans*, aiming to further evaluate the therapeutic potential of this compound in the treatment of candidiasis. We then focused specifically on *C. albicans* and *C. parapsilosis*, assessing the effect of 5-FUrd on important virulence factors in both species.

Our results demonstrate that 5-FUrd exhibits significant antifungal activity against a range of *Candida* species: *C. albicans*, *C. glabrata*, *C. parapsilosis*, *C. tropicalis*, *C. krusei*, *C. kefyr*, *C. lusitaniae*, and *C. norvegensis*. An exception is multidrug-resistant *C. auris*, for which the MIC exceeded the highest tested concentration of 5-FUrd (>265 μg/mL). The MIC and MFC values for the other *Candida* species ranged from 0.2 μg/mL to 51.2 μg/mL. Among them, *C. albicans* and *C. parapsilosis* were the most susceptible, with MIC values of 0.4 μg/mL and 0.2 μg/mL, respectively. The MFC/MIC ratios observed for all the *Candida* species indicate that 5-FUrd is both fungistatic and fungicidal, suggesting its potential as an effective therapeutic agent. Interestingly, 5-FUrd exhibited variable activity against a panel of 100 clinical strains of *C. albicans*. Most strains were highly susceptible to 5-FUrd, with MIC values ranging from 0.1 to 6.4 μg/mL, although some showed relatively high MICs (≥25.6 μg/mL). This variability underscores the need for further studies on the mechanisms of resistance to 5-FUrd, which may be crucial for optimizing its clinical application.

5-FUrd is particularly effective against *C. albicans* and *C. parapsilosis.* While *C. albicans* remains the leading cause of nosocomial invasive candidiasis worldwide, infections caused by non-albicans *Candida* species (NACs), including *C. parapsilosis*, have been increasingly reported. *C. parapsilosis* poses a considerable risk to immunocompromised individuals, including patients with HIV and those undergoing surgical procedures, particularly involving the gastrointestinal tract. Importantly, *C. parapsilosis* accounts for approximately one-third of neonatal *Candida* infections and is associated with a mortality rate of about 10% [25,26].

*Candida* species have developed several specific and effective strategies to enhance their pathogenicity, including cell adhesion, biofilm, formation of hyphae, secretion of hydrolytic enzymes, and hemolytic activity. Virulence factors have also been considered as potent antifungal targets [27,28]. Biofilm formation by *C. albicans* and *C. parapsilosis* plays a crucial role in their pathogenicity. They form biofilms not only on implanted medical devices, including catheters, pacemakers, heart valves, joint prostheses, and dentures, but also on host surfaces, such as mucosal membranes, epithelial cell linings, and parenchymal organs. Biofilms are structured microbial communities embedded in the extracellular matrix (ECM). Biofilm development proceeds through three regulated stages: initial adhesion, an intermediate phase with yeast-to-hyphae transition and multilayer formation, and a maturation/dispersion phase. Biofilm architecture, morphology, and hyphae vary among *Candida* species and even between strains [29]. *C. albicans* generally forms larger and more complex biofilms than other *Candida* species, characterized by a heterogeneous structure composed of yeast cells, hyphae, and pseudohyphae embedded in the extracellular matrix (ECM). The ECM of *C. albicans* biofilms includes proteins and their glycosylated forms, carbohydrates, lipids, and extracellular DNA [30]. In contrast, *C. parapsilosis*, which does not produce true hyphae, forms biofilms consisting mainly of aggregated blastoconidia and pseudohyphae, resulting in a lower biofilm volume compared to other *Candida* species. Additionally, the ECM of *C. parapsilosis* biofilms is predominantly composed of carbohydrates, with relatively low protein content [25]. Our results show that 5-FUrd has a significant impact on the biofilm structure and composition in both *Candida* species. The compound reduced biofilm biomass and metabolic activity in both species during all biofilm formation phases: adhesion, biofilm formation, and even maturation. Additionally, the reduction in the extracellular matrix components, including polysaccharides, proteins, and extracellular DNA in *C. albicans* or polysaccharides and proteins in *C. parapsilosis*, indicates that 5-FUrd may interfere with biofilm formation through multiple pathways. Notably, the extracellular DNA in the *C. albicans* matrix is significantly reduced, which is probably associated with the intracellular activity of 5-FUrd disrupting DNA synthesis. In a study conducted by Martins et al. (2012), the reduction in extracellular DNA by the addition of DNase improved the anti-biofilm activity of some antifungal drugs [31]. This suggests that 5-FUrd may not only inhibit fungal growth but also directly target biofilm structure, which is a promising feature for combating persistent fungal infections.

Adhesion is a critical multifactorial process mediated by both fungal and host cell characteristics, including cell surface hydrophobicity, cell wall composition, and growth conditions. Initially, yeast cell adhesion relies largely on hydrophobic interactions between the microorganism and the host surface. Cell surface hydrophobicity (CSH) is also strongly correlated with adhesion to abiotic surfaces. *Candida* species generally exhibit high levels of CSH [32]. The slight reduction in CSH observed in both *C. albicans* and *C. parapsilosis* following the exposure to 5-FUrd suggests that this compound disrupts key surface properties involved in the host–pathogen interaction, potentially reducing the ability of yeast cells to adhere and form mature biofilms.

The ability to switch between yeast and hyphal growth forms (dimorphism) is one of the most discussed and best investigated virulence attributes of the human pathogenic *Candida* fungi. The inhibition of hyphal formation is considered a potential therapeutic strategy [33]. Several small molecules, including farnesol, fatty acids, rapamycin, geldanamycin, histone deacetylase inhibitors, and cell cycle inhibitors, have been shown to modulate the yeast-to-hyphae transition in *C. albicans*. Additionally, some established antifungal agents, such as azoles, are also known to specifically inhibit hyphal growth [34]. The impact of 5-FUrd on the morphology of *C. albicans* and *C. parapsilosis* was also evaluated in the present study. In *C. albicans*, the compound inhibited hyphal formation in both liquid and solid culture conditions. In contrast, *C. parapsilosis* exhibited a more limited morphological response. Fewer pseudohyphal cells were observed in liquid cultures, while no significant changes were noted in colonies grown on solid media, which did not produce hyphae even in the control conditions (without 5-FUrd). These findings suggest that 5-FUrd exerts a more pronounced morphological effect on *C. albicans* than on *C. parapsilosis*, indicating species-specific differences in sensitivity to the compound.

The secretion of extracellular enzymes, such as aspartyl proteases, lipases, and phospholipases, is a well-established virulence factor in *Candida* species, closely associated with adhesion, host cell damage, and tissue invasion [32]. The production of hemolytic factors by pathogenic *Candida* species is also considered a key mechanism for survival within the mammalian host, enabling iron acquisition from the hemoglobin–heme complex. Free hemoglobin is an important host factor triggering the yeast cell differentiation pathway necessary for the dissemination and establishment of superficial infections. In addition, hemolytic activity may facilitate hyphal invasion in systemic candidiasis [35,36]. Compared to *C. albicans*, *C. parapsilosis* exhibits lower protease and hemolytic activity, and the role of phospholipases in *C. parapsilosis* remains poorly understood [25,37]. Our results showed that 5-FUrd did not inhibit phospholipase activity in *C. albicans* and *C. parapsilosis*, indicating that its antifungal mechanism may not involve the direct inhibition of these enzymes. However, the compound slightly reduced both proteolytic and hemolytic activity of *C. albicans* and *C. parapsilosis*, which could contribute to its overall antifungal effects by limiting the degradation of host tissues and thus reducing the invasive potential of the pathogen.

The toxicity of 5-FUrd was evaluated using zebrafish embryos and human erythrocytes. The results showed that 5-FUrd did not cause significant mortality or morphological abnormalities in zebrafish embryos at concentrations up to 8 µg/mL, which corresponds to 20 × MIC for *C. albicans* and 40 × MIC for *C. parapsilosis*. Additionally, no changes in the heart rate or embryonic development were observed, suggesting that the compound is not toxic to zebrafish at therapeutic concentrations. Similarly, the compound caused minimal erythrocyte lysis, with less than 10% destruction of human red blood cells, indicating that 5-FUrd is safe for human erythrocytes at high concentrations. These findings support the potential of 5-FUrd as an antifungal agent with a favorable safety profile.

Spontaneous mutation frequencies were evaluated to assess the potential for resistance development. The results showed that 5-FUrd caused a moderate increase in the mutation frequency in both *C. albicans* and *C. parapsilosis*, which is typical of antifungal agents that interfere with nucleic acid synthesis, such as ribavirin [38]. However, this increase was relatively higher than that observed with classical antifungals, such as rezafungin [39]. These mutations led to a ≥4-fold shift in MIC values, indicating a potential for resistance development, albeit at relatively low frequencies. These findings suggest that 5-FUrd should preferably be used for a short time or in combination with other antifungal agents. Therefore, beyond the discovery of novel antifungals, a pragmatic strategy would be to enhance the efficacy of existing drugs through combination therapy. In this context, testing 5-FUrd in combination with conventional antifungals in vitro could be highly beneficial for broadening the antimicrobial spectrum, minimizing the emergence of resistance, and reducing side effects by allowing the use of lower effective drug concentrations.

## 4. Materials and Methods

### 4.1. Fungal Strains, Compounds, and Growth Conditions

The reference strains of *Candida* (*C. albicans* ATCC 10231, *C. auris* ATCC MYA-5001, *C. parapsilosis* ATCC 22099, *C. glabrata* ATCC 15126, *C. krusei* ATCC 14243, *C. tropicalis* ATCC 13803 *C. lusitaniae* ATCC 34449, *C. kefyr* ATCC 204093, and *C. norvegensis* ATCC 22977) were obtained from the American Type Culture Collection (ATCC, Gaithersburg, MD, USA). A total of 100 clinical isolates of *C. albicans* were obtained as stock cultures from the Jan Boży Independent Public Provincial Hospital in Lublin, Poland. The strains were identified using VITEK 2 YST IC CARDS (bioMérieux, Warsaw, Poland). The yeast were routinely grown in YPD (1% yeast extract, 2% peptone, 2% glucose) liquid medium at 30 °C with agitation (200 rpm). Sabouraud Dextrose (Biocorp, Warsaw, Poland) and RPMI-1640 medium (Sigma-Aldrich, St. Louis, MO, USA) buffered with 0.165 M morpholinepropanesulfonic acid (Sigma-Aldrich, St. Louis, MO, USA) to a pH of 7.0 were used as well. 5-fluorouridine (5-FUrd), dimethyl sulfoxide (DMSO), 3-(4,5-dimethylthiazol-2-yl)-2,5-diphenyltetrazolium bromide (MTT), menadione, crystal violet (CV), fetal bovine serum (FBS), phosphate-buffered saline (PBS), octane, and other chemicals were purchased from Sigma-Aldrich (Sigma-Aldrich, St. Louis, MO, USA). Human blood for hemolytic tests was purchased from Biomaxima (Lublin, Poland).

### 4.2. Antifungal Susceptibility Testing

The antifungal activity of 5-fluorouridine (5-FUrd) against yeast strains was evaluated using the broth microdilution method according to the Clinical and Laboratory Standards Institute (CLSI) guidelines [40], as described by Khabnadideh et al. (2012) [41]. Serial dilutions of 5-FUrd (0.1–256 µg/mL) were prepared in 96-well microtiter plates using RPMI-1640 medium buffered with MOPS. Stock inocula were prepared by suspending three colonies of each tested yeast strain in sterile 0.85% NaCl, followed by adjusting the turbidity to the 0.5 McFarland standard. A working suspension was then prepared by performing a 1:1000 dilution of the stock suspension in RPMI-1640 medium. After dispensing 0.1 mL of the inoculated suspension into each well, the microtiter plates were incubated at 37 °C for 24 h. Uninoculated medium was included as a sterility control (blank). The minimum inhibitory concentration (MIC) was defined as the lowest concentration of 5-FUrd that inhibited visible growth of the microorganism. Minimal fungicidal concentrations (MFCs) were determined by subculturing aliquots from the wells corresponding to the MIC, 2 × MIC, 4 × MIC, and 8 × MIC onto Sabouraud Dextrose Agar plates (without 5-FUrd). Specifically, 10 μL from each relevant well was plated and incubated at 37 °C for 48 h. The MFC was defined as the lowest concentration of the compound that prevented visible fungal growth on solid medium.

### 4.3. Cell Adhesion

In a 96-well flat-bottom microtiter plate, *C. albicans* or *C. parapsilosis* cell suspensions (2.5 × 10^5^ CFU/mL) in the RPMI-1640 medium were added to the wells containing 5-FUrd at concentrations in the range of 0–51.2 μg/mL (100 μL per well). The plates were incubated at 37 °C for 2 h to allow initial yeast adherence. Subsequently, the wells were washed with phosphate-buffered saline (PBS) to remove loosely bound cells. Fresh medium was then added, and the plates were incubated for 24 h at 37 °C. After triple washing with PBS, the resulting biofilm was treated with 40 µL of MTT (1 mg/mL), 2 µL of 0.4 mM menadione, and 158 µL of PBS in order to determine the viability of the biofilm. After incubation at 37 °C for 3 h, absorbance at 490 nm was measured using a microplate reader (Biotek Synergy HT, Winooski, VT, USA).

Identically prepared *C. albicans* and *C. parapsilosis* biofilms treated with 5-FUrd were stained with crystal violet (CV) to determine total biofilm biomass. For this quantification, each well was washed three times with sterile PBS, and the remaining biofilms were dried at 65 °C for 2–4 h. Then, 100 μL of a 0.5% (*w*/*v*) CV solution was added to each well, and the plates were incubated at room temperature for 20 min. Excess dye was removed by thoroughly rinsing the plates with water. The plates were then air-dried at room temperature for 24 h. CV bound to the biofilm biomass was solubilized by adding 200 μL of methanol. After 20 min incubation at room temperature, absorbance was measured at 570 nm using a spectrophotometric reader. The percentage of biofilm eradication was calculated using the following formula:

% Eradication = [(OD growth control − OD sample)/OD growth control] × 100

### 4.4. Effect of 5-FUrd on Yeast Biofilm Formation and Preformed Biofilms

The biofilm formation assay was performed in 96-well microtiter plates. The cell suspension was prepared in RPMI-1640 medium at a final density of 1 × 10^6^ cells/mL, and 100 μL was dispensed into each well of microtiter plates. Serially double-diluted concentrations of 5-FUrd in RPMI-1640 medium were added to the wells. In the control, 100 μL of RPMI-1640 medium containing 1% DMSO without 5-FUrd was added to selected wells. The plates were then incubated at 37 °C for 24 h.

To assess the effect of 5-FUrd on preformed biofilms, the initial biofilm formation was induced by dispensing 100 μL of a yeast cell suspension (1 × 10^6^ cells/mL in RPMI-1640 medium) into each well of a microtiter plate, followed by incubation at 37 °C for 24 h. After incubation, the medium was aspirated, and non-adherent cells were removed by washing the wells three times with sterile phosphate-buffered saline (PBS). Subsequently, 100 μL of serial two-fold dilutions of 5-FUrd in RPMI-1640 were added to the pre-formed biofilms. The control wells received 100 μL of RPMI-1640 medium containing 1% DMSO without the tested compound. The plates were further incubated at 37 °C for another 24 h. The metabolic activity of biofilms was evaluated using the MTT assay, following the procedure described previously. Total biofilm biomass was quantified by crystal violet staining, also according to the previously described protocol.

### 4.5. Microscopic Analysis of Biofilms

*C. albicans* and *C. parapsilosis* biofilms were grown on poly-L-lysine (PLL)-coated glass microscope coverslips. Clean coverslips were immersed in a 0.1% PLL solution for 10 min, air-dried overnight, rinsed with sterile deionized water, and air-dried again. Overnight yeast cultures grown in YPD medium were diluted to a final concentration of 1 × 10^6^ cells/mL in RPMI-1640 medium supplemented with 10% fetal bovine serum (FBS). Subsequently, 20 mL of the cell suspension was transferred into sterile Petri dishes containing either 5-FUrd at concentrations of 0.4 µg/mL and 12.8 µg/mL or 1% DMSO as a control. The samples were incubated for 24 h at 37 °C without agitation. After incubation, the coverslips were gently rinsed with sterile water to remove non-adherent (planktonic) cells. Biofilms were then stained with crystal violet and visualized using a light microscope.

### 4.6. Extraction and Chemical Composition Analysis of Extracellular Polymeric Substances (EPS)

The extracellular polymeric substances (EPS) of *C. albicans* and *C. parapsilosis* biofilms were extracted using a sonication-based method, followed by analysis of DNA, protein, and carbohydrate content [42,43]. Biofilms were formed in 96-well flat-bottom microtiter plates. Cell suspensions of *C. albicans* or *C. parapsilosis* (2.5 × 10^5^ CFU/mL) in RPMI-1640 medium were dispensed into wells containing 5-FUrd at final concentrations of 6.4 μg/mL and 25.6 μg/mL (100 μL per well). The plates were incubated at 37 °C. After 24 h and 48 h incubation, the supernatants were aspirated, and the remaining adherent cells were gently washed with sterile phosphate-buffered saline (PBS). The biofilms were then suspended in 1 mL of a 0.01 M KCl solution and collected by vortexing and scraping. Yeast biofilm cells were dispersed using a sonicator (Sonic Ruptor 250, Omni International, The Homogenizer Company, Kennesaw, GA, USA) in six cycles of 5 s operation and 5 s pause at a power level of 20 kHz [44,45]. The sonicated suspension was pelleted by centrifugation for 10 min at 7000× *g* at 4 °C. The supernatants were collected and filtered using a 0.22 mm membrane filter (Genoplast Biotech S. A., Rokocin, Poland). The DNA content was quantified using the Quant-iT™ PicoGreen^®^ dsDNA Assay Kit (Thermo Fisher Scientific, Warsaw, Poland). The protein concentration was determined using the Lowry method with the use of the Pierce™ Modified Lowry Protein Assay Kit (Thermo Fisher Scientific, Warsaw, Poland). The carbohydrate content was measured with the phenol-sulfuric acid method, using glucose as a standard [46]. The quantities of these three EPS components were assessed at both 24 h and 48 h of biofilm maturation. Each experiment was performed in at least three independent replicates.

### 4.7. Cell Surface Hydrophobicity (CSH) Assay

The cell surface hydrophobicity of *Candida* cells was determined using the water–hydrocarbon two-phase assay [47]. Cell suspensions (2 mL, 1 × 10^6^ CFU/mL) supplemented with 5-FUrd at 0.05 μg/mL, 0.1 μg/mL, and 0.2 μg/mL against *C. albicans* or 0.025 μg/mL, 0.05 μg/mL, and 0.1 μg/mL against *C. parapsilosis* were incubated in glass tubes for 18 h at 37 °C. Next, the suspensions were centrifuged for 10 min at 3000× *g*, and the fungal cells were resuspended with sterile PBS at OD_600_ of 1.0. An aliquot of the strain solution (1.2 mL) was transferred into a clean glass tube and octane was added (0.3 mL). The solutions were vigorously vortexed for 3 min and separated into two distinct phases. The OD_600_ of the aqueous phase was determined. The control was set as the OD_600_ of the aqueous phase without the octane overlay. The relative hydrophobicity was expressed as the percentage change in the optical density (OD_600_) of the untreated cells (100%).

### 4.8. Effect of 5-FUrd on Cell and Colony Morphology

The effect of 5-FUrd on the morphology of cells and colonies of *C. albicans* and *C. parapsilosis* was evaluated using both liquid and solid RPMI-1640 medium containing 10% fetal bovine serum (FBS). To assess morphological changes in planktonic yeast cells, the cultures were incubated with 5-FUrd at concentrations of 0.1 μg/mL and 0.2 μg/mL for *C. albicans* and 0.05 μg/mL and 0.1 μg/mL for *C. parapsilosis*. Cells were grown at 37 °C for 10 h in a shaking incubator. After incubation, 10 mL aliquots of the cultures were centrifuged, and the resulting cell pellets were resuspended in phosphate-buffered saline (PBS). The cell suspensions were adjusted to 1 × 10^4^ cells/mL and gently applied onto microscope slides. The cells were stained with crystal violet and observed under a light microscope to evaluate morphological alterations (Mikrolab, Warsaw, Poland).

For assessment on solid media, the *Candida* cell suspensions (1 × 10^7^ cells/mL in PBS; 100 μL) were plated onto RPMI-1640 agar containing 10% FBS and supplemented with 5-FUrd at the same concentrations used for liquid cultures. Control plates lacking 5-FUrd served as a reference. The plates were incubated at 37 °C for 24 h. Representative colonies were examined and compared to those on the control plates using an inverted microscope imaged with a digital camera (Mikrolab, Warsaw, Poland).

### 4.9. Phospholipase Activity

The phospholipase activity of *C. albicans*, *C. parapsilosis*, and *C. glabrata* (used as a negative control) was determined using the egg yolk agar plate method [48]. The egg yolk agar medium was prepared by enriching Sabouraud Dextrose Agar (13 g) with NaCl (11.7 g), CaCl_2_ (0.11 g), and 10% sterile egg yolk in a final volume of 184 mL of distilled water. The yeast cells were incubated in the presence of 5-FUrd at concentrations of 0.05 μg/mL, 0.1 μg/mL, and 0.2 μg/mL for *C. albicans* and 0.025 μg/mL, 0.5 μg/mL, and 0.1 μg/mL for *C. parapsilosis*. After 20 h incubation at 37 °C with shaking, the cells were harvested by centrifugation and diluted in sterile PBS. After centrifugation for 10 min at 3000× *g*, the cell pellets were resuspended in sterile PBS. The final cell suspension was adjusted to 1 × 10^8^ cells/mL. Then, 10 μL of each standardized *Candida* suspension was inoculated onto the surface of the egg yolk agar and allowed to dry at room temperature. The plates were then incubated at 37 °C for 48 h. Phospholipase activity was assessed by observing the precipitation zone around the colonies, indicative of enzyme production. The phospholipase activity was quantified using the phospholipase index (Pz) calculated as Pz = *a*/*b*, where *a* is the diameter of the colony plus the precipitation zone, and *b* is the diameter of the colony alone. Phospholipase activity was categorized as follows: very high (Pz ≤ 0.69), high (Pz = 0.70–0.79), low (Pz = 0.80–0.89), very low (Pz = 0.90–0.99), and negative (Pz = 1) [49]. All experiments were performed in triplicate.

### 4.10. Proteinase Secretion

The effect of 5-FUrd on proteinase secretion by *C. albicans* and *C. parapsilosis* was evaluated using a casein degradation assay, following the method described by Ramesh et al. (2011) [50]. Yeast cells were cultured under 5-FUrd pressure using the same concentrations and incubation conditions as described above. Cell-free supernatants (1 mL) were mixed with 1 mL of a casein solution prepared in phosphate buffer (pH 7.5) and incubated at 37 °C for 1 h. The reaction was stopped by adding 0.2 M trichloroacetic acid (TCA). Then, the reaction mixture was centrifuged at 3000 rpm for 10 min. The supernatant was then combined with 0.55 M sodium carbonate and Folin–Ciocalteu reagent and incubated for 15 min at room temperature. Optical density (OD) was measured at 650 nm against the control. The proteolytic activity in the control sample (cultured without 5-FUrd) was defined as 100%. The relative reduction in enzyme activity in the presence of 5-FUrd was expressed as a percentage of the control. All experiments were performed in triplicate.

### 4.11. Hemolytic Factor Secretion

*Candida* cells were cultured in Sabouraud Dextrose Broth at 37 °C for 18 h in the presence of 5-FUrd at 0.05 μg/mL, 0.1 μg/mL, and 0.2 μg/mL for *C. albicans* and 0.025 μg/mL, 0.5 μg/mL, and 0.1 μg/mL for *C. parapsilosis*. Untreated cells cultured in the same conditions served as the control. Then, the cultures were harvested by centrifugation at 1000 × *g* for 15 min and washed with sterile phosphate-buffered saline (PBS). The yeast cells were resuspended in RPMI-1640 medium supplemented with 3% glucose at a final concentration of 1 × 10^4^ cells/mL and incubated at 37 °C for 48 h with shaking at 180 rpm. After incubation, the cultures were centrifuged again at 1000× *g* for 15 min, and the supernatants were concentrated tenfold using centrifugal filters (VWR International, USA). The concentrated culture supernatants were mixed at a 1:1 (*v*/*v*) ratio with a suspension of erythrocytes (1 × 10^8^ cells/mL) prepared in RPMI 1640 medium. The mixtures were incubated at 37 °C for 18 h. A 1% Triton X-100 solution was the positive control (representing 100% hemolysis), while a mixture of erythrocytes and RPMI 1640 medium with PBS (1:1, *v*/*v*) served as the negative control. After incubation, the samples were centrifuged at 1000× *g* for 2 min, and the absorbance of the supernatants was measured at 405 nm. The inhibition of hemolysis by 5-FUrd was calculated as a percentage in relation to the control (untreated cells) [51].

### 4.12. Frequency of Spontaneous Mutations

The frequency of spontaneous mutations was determined in *C. albicans* ATCC 10231 and *C. parapsilosis* ATCC 22099. One hundred microliters of yeast suspensions were spread onto Sabouraud Dextrose Agar (SDA) plates containing 5-FUrd at concentrations corresponding to MIC, 2 × MIC and 4 × MIC. After 24 h of incubation at 37 °C, the frequency of spontaneous mutations was calculated by dividing the number of resistant colonies observed on each drug-containing plate by the number of colony-forming units (CFUs) in the initial inoculum (2.5 × 10^5^ CFU/mL). To determine the initial viable count, serial dilutions of the starting inoculum were prepared and plated in triplicate onto SDA plates without 5-FUrd. Resistant phenotypes were confirmed by re-isolation of mutant colonies and subsequent determination of their MIC values [38].

### 4.13. Hemolytic Assay

The hemolytic activity of 5-FUrd was determined using human red blood cells. Erythrocytes were harvested by centrifugation at 2000 rpm for 10 min at 20 °C and washed three times with phosphate-buffered saline (PBS). The erythrocyte pellet was resuspended in PBS to prepare a 10% (*v*/*v*) erythrocyte suspension. This suspension was further diluted 1:10 with PBS. A volume of 450 μL of the diluted erythrocyte suspension was mixed with 50 μL of PBS containing a predetermined 5-FUrd concentration gradient (ranging from 0.4 to 8 μg/mL) in microcentrifuge tubes. Total hemolysis was induced using 1% Triton X-100 as a positive control. The tubes were incubated at 37 °C for 1 h, followed by centrifugation at 2000 rpm for 10 min at room temperature. Then, 150 μL of the supernatant was transferred to a flat-bottom microtiter plate, and absorbance was measured spectrophotometrically at 450 nm. The percentage of hemolysis was calculated using the following equation:

% Hemolysis = [(A_450_ of 5-FUrd-treated sample − A_450_ of PBS control)/(A_450_ of 1% Triton X-100 − A_450_ of PBS control)] × 100

### 4.14. Toxic Effects of 5-FUrd on Zebrafish Embryos

To determine the toxicity of 5-FUrd, a Fish Embryo Toxicity (FET) test was performed on zebrafish (*Danio rerio*) according to OECD Test Guideline 236 [52,53]. Fertilized zebrafish embryos were exposed to 5-FUrd for 96 h at concentrations ranging from 0.4 µg/mL to 8 µg/mL. The E3 solution (5 mM NaCl, 0.33 mM MgCl_2_, 0.33 mM CaCl_2_, 0.17 mM KCl; pH 7.2) was used as the embryo culture medium and to prepare the solutions. The experiments were conducted in 24-well plates, with ten embryos per well, in triplicate. The plates were covered and incubated at 28 °C. At the end of the 96 h exposure period, acute toxicity was assessed based on a positive outcome in any of the four visual indicators of lethality: coagulation of fertilized eggs, lack of somite formation, failure of the tailbud to detach from the yolk sac, and absence of heartbeat. The mortality rate was expressed as the percentage of dead embryos. The heart rate was monitored using a stopwatch and direct microscopic observation. Zebrafish embryo development and viability were evaluated using bright-field microscopy (Zeiss Stereo Discovery V8, ZEISS, Germany). Image analysis was performed using Zeiss ZEN lite to determine the percentage of dead and malformed embryos over time.

### 4.15. Statistical Analysis

All data are expressed as a mean ± SD (standard deviation) of three independent experiments. Statistical significance between the treated and control groups was analyzed by Student’s *t*-test using GraphPad Software version 9.1.1. (San Diego, CA, USA). *p* value < 0.05 was considered statistically significant.

## 5. Conclusions

5-FUrd exhibits potent antifungal activity against *C. albicans* and *C. parapsilosis*, effectively reducing fungal growth and impairing crucial virulence factors, including adhesion, biofilm formation, hyphal development, and both proteolytic and hemolytic activity. These effects suggest a multi-target mode of action that interferes with fungal pathogenicity. This alternative antifungal strategy, which focuses on targeting virulence factors rather than exclusively essential growth processes, represents a promising avenue for the antifungal activity of 5-FUrd. Additionally, targeting the pathogen-specific virulence mechanisms may help preserve the host’s normal commensal microbiota. Toxicity assays confirm the safety of 5-FUrd used at therapeutic concentrations although the moderate increase in the mutation frequency indicates potential for resistance development. Therefore, short-term use or combination therapy is recommended. Overall, 5-FUrd represents a promising antifungal candidate warranting further investigation.

## Figures and Tables

**Table 1:** MICs (μg/mL) and MFCs (μg/mL) of 5- FUrd against *Candida* species.

*Candida* Species	MIC (μg/mL)	MFC (μg/mL)	MFC/MIC Ratio	Activity
*C. albicans* ATCC 10231	0.4	0.4	1	FST and FCD
*C. auris* ATCC MYA-5001	>256	n.t.	n.t.	n.a.
*C. glabrata* ATCC 15126	12.8	12.8	1	FST and FCD
*C. krusei* ATCC 14243	6.4	12.8	2	FST and FCD
*C. parapsilosis* ATCC 22099	0.2	0.2	1	FST and FCD
*C. tropicalis* ATCC 13803	51.2	51.2	1	FST and FCD
*C. lusitaniae* ATCC 34449	51.2	51.2	1	FST and FCD
*C. kefyr* ATCC 204093	51.2	51.2	1	FST and FCD
*C. norvegensis* ATCC 22977	25.6	51.2	2	FST and FCD

FST, fungistatic; FCD, fungicidal; n.t., not tested; n.a., no activity.

**Table 2:** Effect of 5-FUrd on phospholipase activity of *C. albicans*, *C. parapsilosis*, and *C. glabrata* (negative control). Pz values depending on the concentration of 5-FUrd.

Strain	5-FUrd Concentration (µg/mL)	Pz Value (Mean ± S.D.)	Phospholipase Activity
*C. albicans*	0 (control)	0.25 ± 0.02	Very high
	0.05	0.27 ± 0.02 ^n.s^	Very high
	0.1	0.27 ± 0.03 ^n.s^	Very high
	0.2	0.27 ± 0.02 ^n.s^	Very high
*C. parapsilosis*	0 (control)	0.33 ± 0.01	Very high
	0.025	0.33 ± 0.02 ^n.s^	Very high
	0.05	0.33 ± 0.03 ^n.s^	Very high
	0.1	0.33 ± 0.02 ^n.s^	Very high
*C. glabrata*	0 (negative control)	1 ± 0.01	Negative

n.s.—statistically non-significant.

**Table 3:** Mutant colony counts and spontaneous frequencies for 5-FUrd.

Strain	5-FUrd Concentration (µg/mL)	Plate	No. of Colonies	Frequency	MIC Range (µg/mL)
	0.4	1	26	1 × 10^−4^	1.6–6.4
		2	22	8.8 × 10^−5^	
		3	31	1.2 × 10^−4^	
		4	15	6 × 10^−5^	
		5	19	7.6 × 10^−5^	
		6	21	8.4 × 10^−5^	
*C. albicans*	0.8	1	0	-	-
		2	0	-	-
		3	0	-	-
		4	0	-	-
		5	0	-	-
		6	0	-	-
	1.6	1	0	-	-
		2	0	-	-
		3	0	-	-
		4	0	-	-
		5	0	-	-
		6	0	-	-
*C. parapsilosis*	0.2	1	29	1.2 × 10^−4^	0.8–8
		2	27	1.1 × 10^−4^	
		3	22	8.8 × 10^−5^	
		4	25	1 × 10^−4^	
		5	26	1 × 10^−4^	
		6	23	9.2 × 10^−5^	
	0.4	1	21	8.4 × 10^−5^	1.6–8
		2	19	7.6 × 10^−5^	
		3	25	1 × 10^−4^	
		4	27	1.1 × 10^−4^	
		5	22	8.8 × 10^−5^	
		6	17	6.8 × 10^−5^	
	0.8	1	7	2.8 × 10^−5^	8–32.4
		2	5	2 × 10^−5^	
		3	11	4.4 × 10^−5^	
		4	5	2 × 10^−5^	
		5	10	4 × 10^−5^	
		6	6	2.4 × 10^−5^	

## Data Availability

The original contributions presented in this study are included in the article. Further inquiries can be directed to the corresponding author.
